# Microscopic Colitis Secondary to Leflunomide: A Case Report

**DOI:** 10.7759/cureus.60064

**Published:** 2024-05-10

**Authors:** Mohamed A Ebrahim, Eli A Zaher, Parth Patel, Daria Zaher

**Affiliations:** 1 Internal Medicine, Ascension Saint Joseph Hospital, Chicago, USA; 2 Internal Medicine, University Clinical Hospital in Bialystok, Bialystok, POL

**Keywords:** histology, colonoscopy, chronic diarrhea, leflunomide, microscopic colitis

## Abstract

Microscopic colitis (MC) is characterized by chronic watery diarrhea that requires histological examination for diagnosis. Here, we present a case of a 63-year-old female with rheumatoid arthritis who developed persistent diarrhea following leflunomide initiation. Despite a normal colonoscopy, random colonic biopsies confirmed MC. Discontinuation of leflunomide led to symptom resolution, implicating it as the causative agent. Leflunomide-induced MC is exceedingly rare, with limited documented cases. Understanding its variability in presentation and timely recognition is crucial. This case underscores the importance of thorough medication history assessment and consideration of drug-induced colitis in patients presenting with unexplained diarrhea, facilitating prompt management and resolution.

## Introduction

Microscopic colitis (MC) is a chronic inflammatory disease of the colon that frequently causes chronic watery diarrhea. Diagnosis relies on the exclusion of other etiologies and histological examination of multiple colonic mucosa biopsy samples, which often show no or only a few abnormalities on endoscopy. MC is believed to be caused by medications, most notably nonsteroidal anti-inflammatory drugs (NSAIDs), proton-pump inhibitors (PPIs), and selective serotonin reuptake inhibitors (SSRIs) [1,2]. 

We depict a case of leflunomide-induced MC, which has been described no more than ten times in the literature [2].

## Case presentation

A 63-year-old female with a history of rheumatoid arthritis presented to the emergency department with complaints of fatigue and diarrhea for the preceding three months. Her diarrhea was described as watery, unrelated to food, and continuous throughout both the day and night. She never had such symptoms before. She likewise denied any fevers, chills, abdominal pain, vomiting, weight loss, bloody or dark stools, changing diet, illicit substance use, or recent travel. Her family history was negative for inflammatory bowel disease and gastrointestinal malignancies. She never had a colonoscopy in the past. Her medication regimen consisted of as needed acetaminophen and leflunomide, which was started two months prior to presentation. 

Vital signs on admission were consistent with tachycardia to 105 bpm but were otherwise normal. Physical examination revealed swelling and stiffness of the wrist and metacarpophalangeal joints bilaterally. There was no presence of abdominal tenderness or guarding. Stool studies were negative for infectious etiologies. Blood workup was consistent with hypokalemia and pre-renal acute kidney injury. Further blood workup for human immunodeficiency virus, thyroid disorders, celiac disease, iron deficiency, and vitamin deficiencies was negative. 

**Figure 1:**
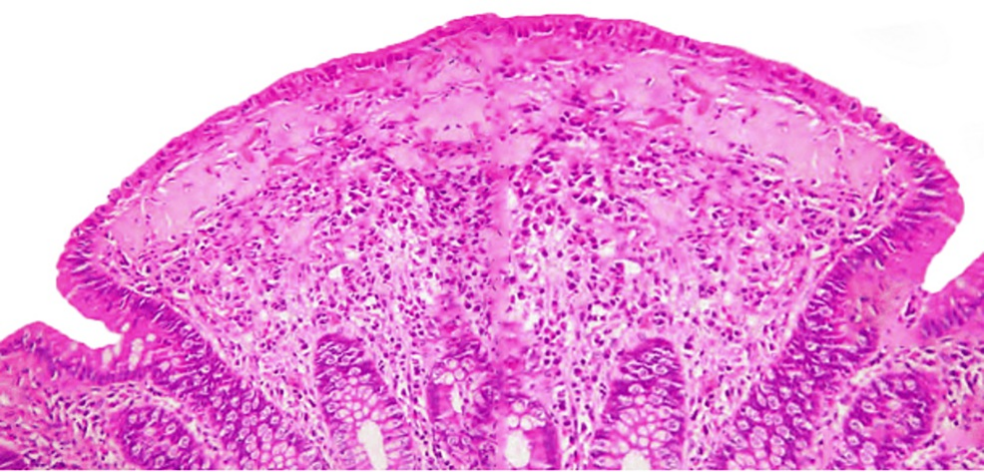
Histological examination of random colonic biopsy - collagenous colitis Prominent thickening of the subepithelial collagen band, characteristic of collagenous colitis.

Our patient was managed conservatively with intravenous hydration and as needed loperamide. Inpatient colonoscopy revealed a normal mucosa. Random colonic biopsies were consistent with microscopic colitis (Figure 1). 

Following a discussion with rheumatology and gastroenterology, our patient opted to discontinue leflunomide. Her symptoms gradually improved and subsequently resolved completely, deeming leflunomide as the culprit. She remains in good health today.

## Discussion

MC represents an inflammatory condition affecting the colon, commonly leading to persistent watery diarrhea, particularly among elderly individuals. Endoscopic examination typically reveals a normal-looking colon in MC cases, necessitating a histological assessment for diagnosis. MC comprises two distinct subtypes: lymphocytic colitis (LC) and collagenous colitis (CC). These subtypes exhibit specific histological characteristics, such as intraepithelial lymphocytosis accompanied by a dense inflammatory infiltrate in the lamina propria, with or without expansion of the subepithelial collagen band [3]. Clinical manifestations of MC are nonspecific and may include chronic or sporadic watery diarrhea, often with nocturnal bowel movements, urgency to defecate, abdominal discomfort, joint pain, and weight loss. In addition, the severity of symptoms varies among individuals [4]. The approximate frequency of MC is estimated to be between one and 25 cases per 100,000 individuals per year [5].

Factors contributing to the risk of MC encompass advancing age, being female, and having concurrent autoimmune conditions. Smoking has been linked to a heightened occurrence of watery stools in MC and an elevated likelihood of persistent disease, with a reduced chance of achieving clinical remission [6]. Drug-induced MC is a potential risk factor, which has varying incidences across populations. While specific medication-related risk factors are not always included in incidence reports, certain drugs have been associated with an increased likelihood of MC development. Notably, PPIs demonstrate a significant correlation with MC, with studies indicating elevated odds ratios for past and current PPI users [7]. Similarly, NSAIDs, particularly aspirin, exhibit heightened risks for MC, emphasizing the importance of NSAID cessation in improving colitis symptoms. SSRIs and angiotensin-converting enzyme inhibitors have also been implicated in MC development, with varying levels of association across studies [8].

Leflunomide, classified as a disease-modifying antirheumatic drug (DMARD), is FDA-approved for the treatment of rheumatoid arthritis. It, an oral medication, transforms into teriflunomide within the body. Teriflunomide inhibits a mitochondrial enzyme called dihydro-orotate dehydrogenase, reducing the synthesis of a nucleotide named ribonucleotide uridine monophosphate pyrimidine (rUMP). This leads to the activation of p53, which blocks cell division from the G1 to S phase, suppressing the proliferation of immune cells. As a result, it exerts anti-inflammatory and immunomodulatory effects. While diarrhea is the primary adverse reaction associated with leflunomide, it usually resolves on its own [9]. Cases of overt colitis triggered by leflunomide are extremely rare, with only a handful reported in the literature, while the precise pathophysiological mechanism remains incompletely understood. Additionally, there appears to be significant variability in how colitis manifests, both in terms of its types and the time elapsed since the initiation of leflunomide treatment [2]. In our case, the patient developed diarrhea two months after leflunomide was initiated for rheumatoid arthritis.

Treatment of MC involves tailoring medical therapies based on symptom severity, treatment response, and medication toxicity. While intermittent courses suffice for many, extended continuous therapy is essential for a minority of patients. Suspending the suspected causative agent could potentially alleviate the symptoms. If persistent, antidiarrheals like loperamide are often the first-line, with high doses considered initially [10]. Bismuth subsalicylate, effective short-term, requires intermittent use due to potential toxicity. Mesalamine, beneficial for induction and maintenance, may be combined with cholestyramine for bile acid malabsorption. Budesonide, well-tolerated and effective, is preferred for active disease and maintenance, especially in CC. Prednisone, less favored due to adverse events, is reserved for refractory cases. Immunosuppressive agents like azathioprine, mercaptopurine, or methotrexate are considered for steroid-resistant or relapsing patients [11]. Surgical intervention, rarely needed, includes diverting ileostomy or proctocolectomy for severe cases unresponsive to medical management.

In all instances of drug-induced MC, including the current one, gastrointestinal symptoms such as diarrhea resolved upon discontinuation of leflunomide, which supported the diagnosis of leflunomide-induced colitis. The average duration until symptom resolution after ceasing leflunomide was approximately three weeks, although it varied from three days to seven weeks. In our patient's case, the time taken for symptoms to resolve was around four weeks, with gradual symptom improvement to complete resolution. It is noteworthy that leflunomide is administered orally and undergoes metabolism in the gastrointestinal tract to its active metabolite, teriflunomide. In patients with rheumatoid arthritis, the half-life of teriflunomide is approximately 15 days, and it is primarily eliminated through urine and feces [8].

## Conclusions

The variation in patient presentations of leflunomide-induced colitis makes establishing definitive diagnostic criteria challenging. This difficulty stems in part from the condition's rarity, highlighting the need for more case reports to better understand typical symptoms. Diagnosis involves ruling out other potential causes of diarrhea, such as infections, inflammation, autoimmune disorders, and hormonal imbalances. A thorough examination of a patient's medication history is crucial, as several drugs can trigger colitis. Improvement in diarrhea after discontinuing leflunomide provides supportive evidence for the diagnosis. Consequently, in patients receiving leflunomide who develop persistent unexplained diarrhea, a colonoscopy with biopsy is recommended, and discontinuation of leflunomide should be highly considered.
